# Oral Ursodeoxycholic Acid Crosses the Blood Retinal Barrier in Patients with Retinal Detachment and Protects Against Retinal Degeneration in an Ex Vivo Model

**DOI:** 10.1007/s13311-021-01009-6

**Published:** 2021-02-03

**Authors:** Alejandra Daruich, Thara Jaworski, Hugues Henry, Marta Zola, Jenny Youale, Léa Parenti, Marie-Christine Naud, Kimberley Delaunay, Mathilde Bertrand, Marianne Berdugo, Laura Kowalczuk, Jeffrey Boatright, Emilie Picard, Francine Behar-Cohen

**Affiliations:** 1 Centre de Recherche des Cordeliers INSERM, UMRS1138, Team 17, Université de Paris, Université Sorbonne Paris Cité, Paris, France; 2 Ophthalmology Department, Necker-Enfants Malades University Hospital, AP-HP, Paris, France; 3 Ophthalmology Department, University of Lausanne, Jules-Gonin Eye Hospital, Fondation Asile des Aveugles, Lausanne, Switzerland; 4 Innovation and Development Laboratory, Clinical Chemistry Service, Lausanne University Hospital, Lausanne, Switzerland; 5 Institut du Cerveau et de la Moelle épinière (ICM), INSERM, CNRS, AP-HP, Sorbonne Université, Pitié-Salpêtrière University Hospital, Paris, France; 6 Ophthalmology Department, Emory University School of Medicine, Atlanta, GA USA; 7 Center of Excellence, Atlanta Veterans Administration Medical Center, Decatur, GA USA; 8 Ophtalmopole, Cochin Hospital, AP-HP, Université de Paris, Paris, France

**Keywords:** Retinal degeneration, retinal detachment, neuroprotection, ursodeoxycholic acid, UDCA, TUDCA

## Abstract

Rhegmatogenous retinal detachment (RD) is a threatening visual condition and a human disease model for retinal degenerations. Despite successful reattachment surgery, vision does not fully recover, due to subretinal fluid accumulation and subsequent photoreceptor cell death, through mechanisms that recapitulate those of retinal degenerative diseases. Hydrophilic bile acids are neuroprotective in animal models, but whether they can be used orally for retinal diseases is unknown. Ursodeoxycholic acid (UDCA) being approved for clinical use (e.g., in cholestasis), we have evaluated the ocular bioavailability of oral UDCA, administered to patients before RD surgery. The level of UDCA in ocular media correlated with the extent of blood retinal barrier disruption, evaluated by the extent of detachment and the albumin concentration in subretinal fluid. UDCA, at levels measured in ocular media, protected photoreceptors from apoptosis and necrosis in rat retinal explants, an ex vivo model of RD. The subretinal fluid from UDCA-treated patients, collected during surgery, significantly protected rat retinal explants from cell death, when compared to subretinal fluid from control patients. Pan-transcriptomic analysis of the retina showed that UDCA upregulated anti-apoptotic, anti-oxidant, and anti-inflammatory genes. Oral UDCA is a potential neuroprotective adjuvant therapy in RD and other retinal degenerative diseases and should be further evaluated in a clinical trial.

**Supplementary Information:**

The online version contains supplementary material available at 10.1007/s13311-021-01009-6.

## Introduction

The neural retina is made up of interneurons and photoreceptors, organized in several layers. The retinal photoreceptor cell layer is adjacent to the retinal pigment epithelium (RPE), a single-cell layer that provides support functions to the retina, recycles components critical to the visual cycle, and interfaces with the choroid, a vascular layer that provides metabolic support to the retina. The retina is attached to the RPE not only through structural factors such as its interaction with photoreceptor segments and extra cellular matrix but also by the active pumping mechanisms of the RPE. Rhegmatogenous retinal detachment (RRD) is a frequent form of retinal detachment (RD) that results from the mechanical tearing of the neural retina by vitreous tractions, leading to subretinal fluid (SRF) accumulation. It affects 13.3 per 100,000 habitants in Europe [1]. RD threatens vision, since photoreceptor cell death begins as early as 12 h after physical separation from the RPE and choroidal vessels [2]. Although reattachment surgery is performed urgently, only two-fifths of patients with RD involving the macula recover 20/40 or better vision [3]. The irreversible loss of function is mainly due to photoreceptor cell death, through mechanisms that recapitulate those occurring in other degenerative retinal diseases [4]. RD is then a human model to test neuroprotective drugs.

A neuroprotective therapy adjuvant to surgery, targeting one or more mechanisms involved in photoreceptor cell death, could improve the visual outcome of patients operated on for RD.

Primary bile acids (BAs) are synthesized from cholesterol in the liver and then excreted into the intestine, where gut microbiota converts primary BA into secondary BA through chemical modifications. The main function of BA is the emulsification, absorption, and digestion of lipids.

However, the hydrophilic secondary BAs, ursodeoxycholic acid (UDCA) and tauroursodeoxycholic acid (TUDCA), the taurine conjugate of UDCA, have also shown neuroprotective effects in neurodegenerative [5] and retinal disease [6]. Recently, TUDCA showed beneficial effects in patients with amyotrophic lateral sclerosis in a randomized controlled clinical trial [5]. In the retina, TUDCA demonstrated anti-apoptotic and anti-oxidant effects in a rat model of RD [7], as well as in other models of photoreceptor degeneration, such as light-induced retinal damage and retinitis pigmentosa [8]. UDCA has shown beneficial effects on vascular integrity, inflammation, and endoplasmic reticulum stress in streptozotocin models of diabetic retinopathy [9, 10], but it has never been evaluated in RD models.

UDCA, unlike TUDCA, is FDA-approved for the treatment of cholesterol gallstone dissolution, and is currently considered the first-choice therapy for several forms of cholestatic syndromes [11]. Moreover, oral UDCA was shown to cross the blood-brain barrier in a dose-dependent manner with excellent tolerability and safety when administered in patients with amyotrophic lateral sclerosis [12]. The ocular bioavailability and tolerance of oral UDCA, however, has not been evaluated in patients with retinal pathology, which is a major limitation for its evaluation in retinal diseases. In the present study, we have measured UDCA levels in the ocular media and more specifically in the SRF of patients who were operated on for RD and treated with oral UDCA before surgery. Next, we tested whether UDCA levels detected in SRF protected retinal explants from cell death, and we explored the mechanisms involved. Finally, we showed that, while SRF from untreated patients was toxic for rat retina explants, SRF from UDCA-treated patients was protective. Through the use of a reverse translational approach, we have brought forth evidence that oral UDCA could be used as an adjuvant therapy and improve retinal detachment surgery outcome.

## Methods

### Clinical Study

This study (ClinicalTrials.gov: NCT02841306) was designed in accordance with the tenets of the Declaration of Helsinki and was approved by the Ethics Committee of the Swiss Federal Department of Health (Authorization CER-VD no. 2016-00644). Written informed consent was received from all participants prior to inclusion. Twenty-six consecutive patients presenting with RRD for more than 4 days from symptom onset were included between the period of July 2016 and September 2016 at Jules-Gonin Eye Hospital, Lausanne, Switzerland. Exclusion criteria included the following: age < 18 years or > 90 years, monophthalmic patients, history of vitrectomy, vitreous bleeding or other associated retinal disease, and any condition or treatment contraindicating the administration of oral UDCA (pregnancy and lactation, peptic ulcer, acute or chronic liver disease, acute infection of the gallbladder and biliary tract, repeated biliary colic, Crohn’s disease, ulcerative colitis, or other disease of the small intestine and colon, galactose intolerance, the Lapp lactase deficiency or glucose and galactose malabsorption, hypersensitivity, treatment by cholestyramine, colestipol, and antacids containing aluminum hydroxide or magnesium, cyclosporine, ciprofloxacin, nitrendipine, or dapsone). Baseline examination included a complete ophthalmological examination, ETDR assessment of best-corrected visual acuity (BCVA), and color fundus photography. Extension of RD was quantified as the number of clock hours of the fundus representation. Blood samples for UDCA determination (400 μL) were collected at baseline (before surgery) and at day 7 after surgery, and were immediately centrifuged (twice at 2000*g*) for plasma extraction and congelation at −80 °C. Blood samples to test hepatic parameters (aspartate aminotransferase, AST (SGOT), alanine aminotransferase, ALT (SGPT), alkaline phosphatase ALP and gamma glutamyl-transpeptidase, γ–GT) were collected at baseline, day 7, monthly for a period of 4 months, and at 6 months after surgery. Oral UDCA (Ursochol®, 300 mg, Zambon Switzerland Ltd., Cadempino, Switzerland) was administered at 10 mg/kg/day before surgery at different intervals (4 groups: ≤5 h, 6–8 h, 9–11 h, or ≥ 12 h), and then after surgery (two-intake/day) during 4 months. Five patients were not treated and served as control group. Standard 23G pars plana vitrectomy, laser or cryotherapy and gas or silicone tamponade, was performed in all patients. Ocular samples were collected during surgery before any surgical repair was undertaken, particularly cryotherapy. All surgeries and samples were performed by the same surgeon (AD) to minimize technical variability. Undiluted vitreous humor (VH) was collected via manual aspiration through a syringe connected to a 23-gauge vitrectomy cutter (before infusion opening; Alcon, Rotkreuz ZG, Switzerland). To collect SRF, the vitrectomy cutter was placed through the retinal tear under the retina before infusion opening, SRF was aspirated manually while infusion was slowly open to avoid hypotony and risk of bleeding. This procedure minimizes vitreous and infusion contamination. Samples were coded and stored in a biobank at −80 °C. Follow-up visits were performed at days 1 and 7, monthly for a period of 4 months, and at 6 months after surgery, and included a complete ophthalmological examination, color fundus photography, spectral-domain optical coherence tomography, microperimetry, and a questionnaire for patient-reported tolerance and occurrence of adverse events (Supplementary Table 1 and 2).

### UDCA and Protein Determination in Ocular and Plasma Samples

Twenty-eight bile acids were measured by isotope-dilution high-performance liquid chromatography coupled to high-resolution mass spectrometry (LC-MS HR). Each sample (50 μL) was combined with the internal standards in methanol (100 μL), and H_2_O with 0.2% formic acid (600 μL) was added. Sample extraction and clean-up were performed using 96-well HLB SPE plates. Bile acids were separated via the method LC gradient within 22 min and were then measured by high-resolution MS. Quantification was based on peak area ratios interpolated against a seven-point calibration curve. The LC-MS HR system (QExactive Orbitrap mass spectrometer) was run using XCalibur 2.2 (Thermo Fisher Scientific, Cergy Pontoise, France). Data files were processed into result files using TraceFinder 3.0 (Thermo Fisher Scientific). Peak areas of target components were realized by auto-integration. The validated result file (TraceFinder 3.0) was exported in an Excel file format. Protein concentration in SRF was calculated using the Micro BCA protein assay (Thermo Fisher Scientific). Total protein concentration in SRF was calculated using the Micro BCA protein assay (Thermo Fisher Scientific).

### Cell Line and *In Vitro* Model

WERI-Rb-1 human cone cell line (HTB-169, ATCC, Manassas, USA) were cultured on Roswell Park Memorial Institute (RPMI)-1640 medium (Thermo Fisher Scientific) and supplemented with 10% fetal bovine serum and 1% penicillin-streptomycin. In order to determine the concentration of albumin needed to induce a significant cell death, WERI-Rb-1 human cones were treated with concentrations of albumin (Bovine Serum Albumin, Sigma-Aldrich Chemical Co., Saint-Quentin en Yvelines, France) from 2.5 to 40 mg/mL (*n* = 4/group) and cultured during 24 h. To determine the neuroprotective effect of UDCA (Sigma-Aldrich), WERI-Rb-1 human cones were treated by 1, 5, and 10 μM of UDCA (*n* = 4–8/group) 1 h earlier to add albumin at 20 mg/mL. After 24 h of culture, 100 μL of incubation medium was collected to determine cytotoxicity by lactate dehydrogenase (LDH) release (Sigma-Aldrich Chemical Co.), as previously described [13]. LDH activity in the incubation medium was compared with that measured after complete lysis of the cells in medium containing 2% Triton X-100. A viability percentage of zero corresponded to 100% LDH activity in the medium. CellTiter (CellTiter 96® AQueous One Cell Proliferation Assay Solution, Promega Corporation, Charbonnières-Les-Bains, France) was used to assess mitochondrial activity. It was added to the cells and then incubated for 2 h. The absorbance was read at 492 nm. The results were calculated on percentage, by reporting the absorbance of cells by group to the mean of the control group.

### Animals and Ex Vivo Models of RD

Adult male Wistar rats (Janvier labs, Le Genest St Isle, France, *n* = 59) were fed with a standard laboratory diet and ad libitum tap water in a room maintained at 21° to 23 °C with a 12-h light/12-h dark cycle (6 a.m. to 6 p.m.) during 7 days, before being sacrificed by carbon dioxide inhalation, following French and European legislation (Décret no. 2013-118 du 1^er^ Février 2013).

Retinal explants were created to mimic retinal detachment conditions. Neuroretinas were dissected from freshly enucleated eyes, separated from the retinal pigment epithelium, divided into two parts, and then transferred to 0.2-mm polycarbonate membranes (Millipore, Saint Quentin En Yvelines, France) with the photoreceptor layer facing up [13]. The membranes were next placed into a six-well culture plate containing Dulbecco’s modified Eagle’s medium (Thermo Fisher Scientific) and 3% fetal bovine serum (3.9 ml/well). Three different models were used: spontaneous cell death (a model of retinal degeneration induced by retinal detachment), albumin-induced cell death, and patient SRF-induced cell death. First, in the spontaneous cell death model, 100 μL of vehicle (control) or UDCA at a final concentration of 10, 50 (concentration rage found in patients), or 500 ng/ml (10 times these concentration range) was added to the photoreceptor side of the explants (upper chamber) (*n* = 4/group). Second, in the albumin-induced cell death model, 100 μL of albumin (bovine serum albumin at 12 mg/ml), alone (control) or containing UDCA at 10 ng/ml was added to the upper chamber (*n* = 5/group). And third, in the SRF-induced cell death model, 100 μL of SRF from patients operated on in standard treatment of retinal detachment was added to the upper chamber. SRF from control (*n* = 5) and SRF from oral UDCA-treated patients (with UDCA found in SRF, *n* = 8) were pooled before being added to the retinal explants. Five groups were compared: SRF from control patients (*n* = 9), SRF from control patients with addition of UDCA at 10 ng/ml (*n* = 5), and SRF from oral UDCA-treated patients (*n* = 5).

The concentration of UDCA and albumin was chosen according to the concentration ranges found in the SRF of patients (except for the ten-time-dose of 500 ng/ml), and serial dilution was performed to reach the final concentrations in the entire volume of the well (4 ml). The dilutions of UDCA or vehicle were successively performed on ethanol 100% (10 mg/ml), phosphate-buffered saline (PBS), and medium (the same prepared for the culture wells). In the albumin-induced cell death model, albumin was added at the last dilution. In the SRF-induced cell death model, the last two dilutions were performed directly within the SRF (and not in medium) (Supplementary Fig. 1). SRF from patients treated or not by oral UDCA was not diluted. Treated explants were cultured 48 h in spontaneous cell death model, and 6 h in the induced cell death models. LDH release was measured in medium collected from the lower chamber. Immunohistochemistry and Western blotting were performed on explants, as described below.

### Western Blotting Analysis

Explants (*n* = 4 or 5 per group) were lysed MPER buffer (Thermo Fisher Scientific) and then centrifuged at 13,000*g* for 5 min at 4 °C. Protein concentrations were calculated using the Micro BCA protein assay (Thermo Fisher Scientific). Five to 10 mg of total extract was then mixed with protein loading buffer (Thermo Fisher Scientific), as per the manufacturer’s instructions. Samples were loaded onto 4 to 12% bis-tris gel (Thermo Fisher Scientific), and proteins were transferred onto nitrocellulose membranes. Nonspecific binding was blocked with 5% nonfat dry milk in Tween/Tris-buffered saline, then membranes were incubated overnight at 4 °C, with the primary antibody against Caspase 3 (1:500; Clone C92-605, BD Transduction Laboratories, CliniSciences, Nanterre, France), receptor-interacting protein (RIP) kinase 1 (1:500; Clone 38, BD Transduction Laboratories), apoptosis-inducing factor (AIF) (E-1) (1:500, Sc-13,116, Santa Cruz Biotechnology, Heidelberg, Germany), or actin (1:4000; Sigma-Aldrich), followed by incubation with the supplier-recommended dilution of horseradish peroxidase–conjugated secondary antibody for 1 h (Vector Laboratories, Eurobio, Les Ulis, France). Protein bands were visualized by an enhanced chemiluminescence reaction (Thermo Fisher Scientific) using a Bioimaging system (MicroChemi 4.2, Berthold, France or Invitrogen iBright Imaging Systems, Thermo Fisher Scientific). The gray values of specific bands were quantified using ImageJ, and the protein signals of interest were reported relative to the actin signal or to their respective cleaved forms (for Caspase 3, AIF) for each sample.

### Immunohistochemistry and Fluorescence Intensity Evaluation

#### Sections of Retinal Explants

Explants were rinsed in 1× phosphate-buffered saline, fixed for 20 min with 4% paraformaldehyde (Inland Europe, Conflans sur Lanterne, France), infiltrated in a sucrose gradient series, and then mounted in Tissue-Tek optimum cutting temperature (OCT) (Siemens Medical, Puteaux, France). Immunohistochemistry was performed on 10-μm-thick sections. Primary anti-rhodopsin antibody (Rho4D2, Abcam, Cambridge, UK) and the corresponding Alexa-conjugated secondary antibody (Thermo Fisher Scientific) were used; sections were counterstained with 4,6-diamidino-2-phenylindole (DAPI, Sigma-Aldrich). Similarly, microglia and macrophages were immunodetected with ionized calcium-binding adapter molecule 1 (IBA1) (Wako Pure Chemical Industries, Neuss, Germany) staining. The sections were photographed with a fluorescence microscope (BX51, Olympus, Rungis, France), using identical exposure parameters for all compared samples. Blind quantifications were realized on photographs acquired at ×40 magnification with ImageJ software. Measurement of the outer segment length was performed in 8 photographs/explant by measuring at three equidistant points on each photograph. The mean was then calculated for each explant (*n* = 5 explant/group). IBA1-positive cells were quantified on photographs acquired at ×20 magnification with ImageJ software, based on their differential shapes (round amoeboid or ramified dendritic form), as previously reported [14], and the results were expressed as a ratio (mean of 8 photographs/explant, *n* = 5 explant/group).

#### Flat-Mounted Retinal Explants

Explants were rinsed in ×1 phosphate-buffered saline, fixed for 20 min with 4% paraformaldehyde, and then permeabilized with PBS/5% Triton/5% fetal bovine serum for 45 min. Incubation with Lectin from *Arachis hypogaea* (1/1000 Peanut Agglutinin, PNA-FITC, Sigma-Aldrich) was performed overnight at 4 °C. DAPI (Sigma-Aldrich) staining was performed by incubation during 20 min. Explants were flat-mounted between a slide and a coverslip, using a mounting medium (Fluoromount, Dako supplied by Agilent Technologies France, Les Ulis, France). The flat-mounted explants were photographed at ×40 magnification with a fluorescence microscope (BX51, Olympus), using identical exposure parameters for all compared samples (6 photographs/explants). Blind quantifications of PNA-positive cells were realized on acquired photographs with ImageJ software.

### Real-Time Reverse Transcription Polymerase Chain Reaction

Cell pellet (*n* = 5 per group) was directly frozen until ribonucleic acid (RNA) isolation with a RNeasy mini kit (Qiagen, Courtaboeuf, France), according to the manufacturer’s protocols. RNA concentration and purity were determined with a NanoPhotometer™ (IMPLEN, Science Tec, Courtaboeuf, France). First-strand cDNA was generated through reverse transcription, using total RNA and a SuperScript IV Reverse Transcription Kit (Thermo Fischer Scientific). Quantitative PCR using duplicate technical replicates was performed on a Quant Studio 5 PCR System (Thermo Fischer Scientific) using SYBR Green gene expression assay probes (QuantiTech Primer Assay, Qiagen) of the human target genes sphingosine-1-phosphate receptor 2 (*S1PR2*, NM_004230; QT00230846), nuclear receptor subfamily 3, group C, member 1 (glucocorticoid receptor, GR) (*NR3C1*, NM_000176; QT00020608), nuclear receptor subfamily 3, group C, member 2 (mineralocorticoid receptor, MR) (*NR3C2*, NM_000901; QT000028490), g protein-coupled bile acid receptor 1 (*GPBAR1*, or Takeda G protein coupled receptor 5 (TGR5), NM_001077191; QT000209594), glyceraldehyde-3-phosphate dehydrogenase (*GAPDH*, NM_001256799; QT00079247), and QuantiFast SYBR Green PCR Master Mix (Thermo Fischer Scientific). The expression levels of individual genes were then normalized with *GAPDH* in the same sample by calculation of the ΔCt value, and relative quantification was performed using the ΔΔCt method, with condition without albumin and UDCA as control.

### Ribonucleic Acid Sequencing and RNA-Seq Data Analysis

Retinas (*n* = 3 per group) were frozen immediately after isolation. Total RNA was extracted using a Precellys homogenizer (Bertin, Montigny-le-Bretonneux, France) and a RNeasy Mini kit (Qiagen). Quality of raw data has been evaluated with FastQC. Poor-quality sequences have been trimmed or removed with Trimmomatic software, in order to retain only good-quality paired reads. Star v2.5.3a has been used to align reads on reference genome using standard options. Quantification of gene and isoform abundances has been done with rsem 1.2.28 prior to normalization on library size with DESEq2 bioconductor package. Finally, differential analysis has been conducted with edgeR bioconductor package. Multiple hypothesis-adjusted *p*-values were calculated with the Benjamini-Hochberg procedure, to control FDR. Log2 fold-change threshold was set to 0.5 and the FDR threshold to 0.05. At least 30% of samples per group respect a minimum CPM threshold of 1. Enrichment analysis has been performed with GSEA (Gene Set Enrichment analysis). Protein-protein interactions have been reconstructed with STRING (Search Tool for the Retrieval of Interacting Genes). The tool was used with a minimum required interaction score of 0.7 and the maximum number of interactors to show was set to 10. The final network connecting the differentially expressed genes with the pathways was obtained with cytoscape.

### Statistics

Results are presented as the mean ± SEM. Analyses were performed using GraphPad (version 5.0, GraphPad Software, La Jolla, CA, USA). Non-normally distributed data was analyzed using a nonparametric Mann-Whitney test, in order to compare two groups. Spearman correlation coefficients were used to investigate association between variables. *p* values <0.05 were considered statistically significant.

## Results

### UDCA Level in Subretinal Fluid Correlates with Protein Concentration and with the Surface Area of the Detached Retina

**Fig. 1:**
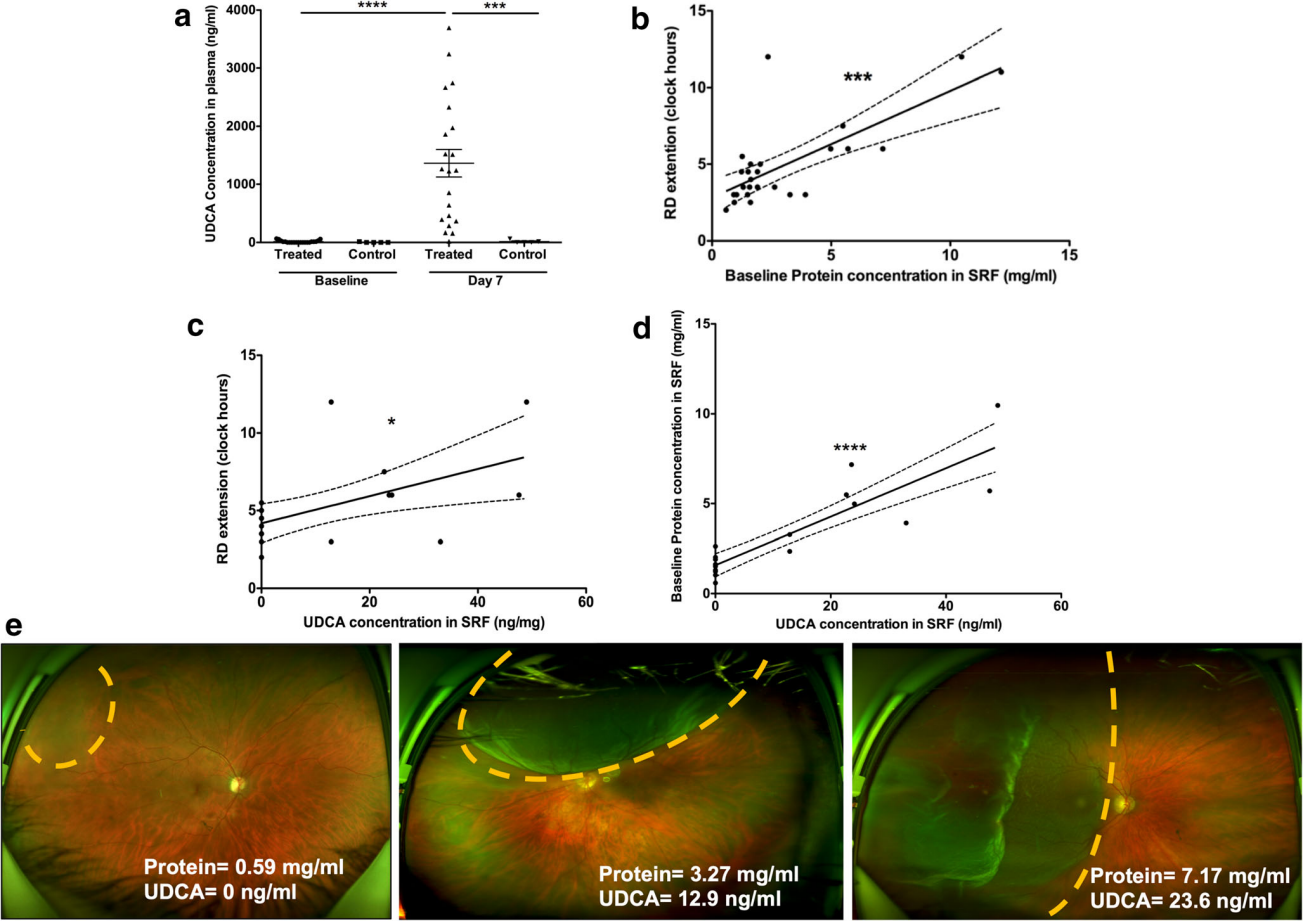
UDCA concentration in plasma and ocular fluids relative to blood retinal barrier rupture in patients with retinal detachment (A) UDCA concentration in plasma at baseline, and at 7 days post-surgery in UDCA-treated patients (*n* = 21) and surgical controls not treated with UDCA (*n* = 5). UDCA concentration in plasma increased significantly only in treated patients at 7 days (****p < 0.0001, Mann-Whitney test). UDCA concentration in plasma at 7 days was significantly higher in UDCA-treated patients, when compared with control (***p = 0.0009, Mann-Whitney test). (B) Baseline protein concentration in SRF was correlated with the extension of RD in clock hours at baseline (*p* = 0.0002, *r* = 0.7, Spearman correlation). (C) UDCA concentration in SRF was correlated with the extension of retinal detachment (RD) in clock hours at baseline (*p = 0.013, *r* = 0.5, Spearman correlation). (D) UDCA concentration in subretinal fluid (SRF) was correlated with protein concentration in SRF at baseline (****p < 0.0001; *r* = 0.8, Spearman correlation). (E) Clinical examples showing the correlation between the extension of RD (dots line), the protein concentration in SRF, and UDCA concentration in SRF

Twenty-six patients with RRD were included (mean age = 62 years, male/female =17/9). All patients presented with simple RRD. Nineteen patients presented with macula off RRD and 7 patients with macula on RRD. Twenty-one patients received oral UDCA at different time intervals prior to surgery (including vitrectomy) for RD repair. Five patients who underwent surgery for RD, but did not receive UDCA, served as controls for ocular fluids (Supplementary Table 1). Ocular fluids, consisting of VH and SRF, were collected separately during surgery. Plasma samples were collected before UDCA was administered and at 7 days of treatment. While plasma UDCA concentration did not change significantly from baseline to 7 days in the control group (from 3.0 ± 6.7 to 15.3 ± 27.0 ng/ml, *p* = 0.6, Mann-Whitney test), UDCA plasma levels increased significantly in the group treated with UDCA at 7 days, as compared to baseline (from 14.6 ± 21.3 at baseline to 1363 ± 1087 ng/ml at 7 days; *p* < 0.0001, Mann-Whitney test) (Fig. 1(A)). UDCA concentration in plasma at 7 days was higher in UDCA-treated patients, compared with control patients (1363 ± 1087 ng/ml vs 15.3 ± 27.0 ng/ml, *p* = 0.0009, Mann-Whitney test) (Fig. 1(A)). UDCA was measured in SRF or VH in 12 of the 21 treated patients (*n* = 4 SRF, *n* = 4 VH, *n* = 4 SRF and VH). The mean concentration in SRF was 28.2 ± 14.0 ng/ml (range = 12.9–49.0 ng/ml), and in VH, it was 19.6 ± 9.9 ng/ml (range = 13.1–40.6 ng/ml). UDCA was detected in one vitreous and in none of SRF of the 5 control patients. UDCA concentration in plasma at baseline did not correlate with the concentration of UDCA in ocular fluids (*p* = 0.43, *r* = 0.2, Spearman correlation). The time interval between oral UDCA intake and ocular fluid sampling (range: 1.8–9.3 h) did not influence the concentration of UDCA in ocular fluids (*r* = 0.1, *p* = 0.29, Spearman correlation).

The extension of blood retinal barrier breakdown was evaluated by the surface area of detached retina at baseline expressed in the number of clock quadrants of the fundus representation and by the protein concentration in the SRF, that were both significantly correlated (*r* = 0.7, *p* = 0.0002, Spearman correlation) (Fig. 1(B)). Mean total protein concentration in SRF was 3.1 ± 2.9 mg/ml (range: 0.6–12.1 mg/ml).

UDCA concentration in SRF significantly correlated with the surface area of detached retina (*r* = 0.5, *p* = 0.013, Spearman correlation), and with protein concentration in SRF (*r* = 0.8, *p* < 0.0001, Spearman correlation) (Fig. 1(C–E)), demonstrating that the ocular bioavailability of UDCA is linked to the breakdown of the outer blood retinal barrier.

Clinical characteristics including change in BCVA at baseline and 6 months after surgery, UDCA levels, and proteins levels in treated and controls patients are reported in Supplementary Table 1 and Supplementary Fig. 2.

UDCA was well-tolerated, and few mild side effects (flatulence, constipation, unpleasant taste) were more frequently reported in UDCA-treated patients (Supplementary Table 2 and 3). Five of 21 patients interrupted treatment before 4 months had passed, due to nausea, epigastric pain, or tingling. Blood hepatic parameters remained within the normal ranges in all patients during the 6-month follow-up.

### UDCA, at Concentrations Found in Ocular Samples of RD Patients, Exerts Neuroprotective Effects in Rat Retinal Explants, an Ex Vivo Model of RD

**Fig. 2:**
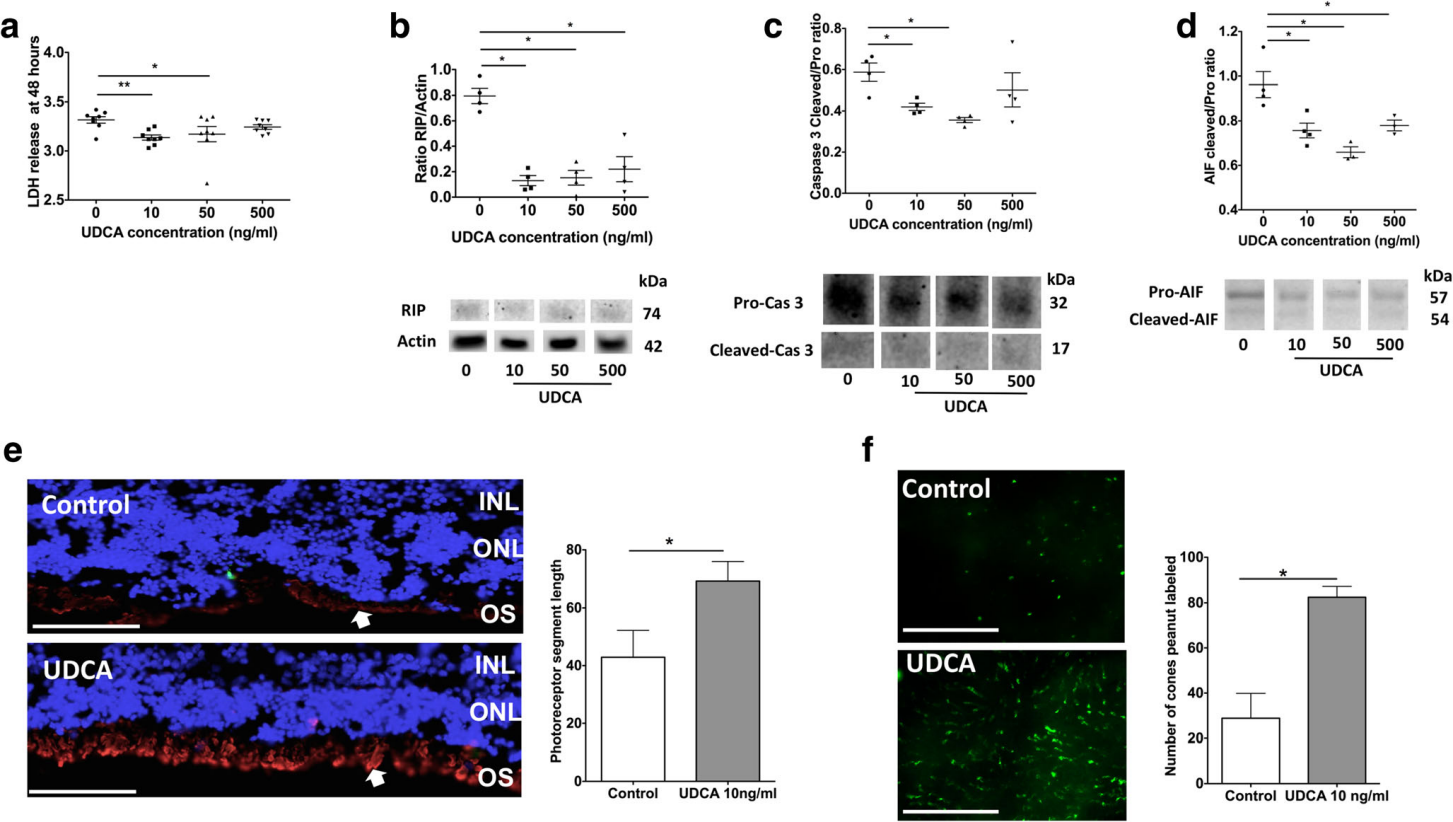
UDCA protects from cell death and preserves photoreceptor in rat retinal explants. Retinal explants were treated with UDCA at concentrations found within patient ocular fluids (10 ng/ml, 50 ng/ml) and at 10 times the highest concentration (500 ng/ml) and cultured for 48 h. Control explants were treated with vehicle. (A) Lactate dehydrogenase (LDH) release was lower in culture medium from retinas treated with UDCA at 10 and 50 ng/ml (**p = 0.002 and *p = 0.046, respectively). (B–D) Western blotting quantification. (B) Receptor-interacting protein (RIP) kinase reported on actin was reduced in UDCA-treated retinas (*p = 0.029). (C) The cleaved form of Caspase 3 reported on the full-form was reduced in retinas treated by UDCA at 10 ng/ml (*p = 0.028) and 50 ng/ml (*p = 0.014). (D) The cleaved form of apoptosis-inducing factor (AIF) reported on full form was lower in UDCA-treated retinas (*p = 0.029). (E) Immunostaining for Rho4D2 (red, arrows) in sections of retina explants. Control and UDCA (10 ng/ml) treated explants (left). Scale: 50 μm. Rod segments length was greater in treated retinas (right) (*p = 0.029). (F) Immunostaining against cone segments (peanut agglutinin, green) on flat-mounted retina explants. Control and UDCA (10 ng/ml)-treated explants (left). Scale: 100 μm. Cone segments number was significantly higher in UDCA-treated retinas (*p = 0.029). Mann-Whitney test

Rat neuroretinas, freshly separated from RPE and placed on membranes with photoreceptors facing up, mimic relevant pathological features of RD such as spontaneous photoreceptor cell death [13]. Rat retinal explants were treated with UDCA at concentrations detected in ocular fluids of treated patients (10 ng/ml, 50 ng/ml), and at 10 times the highest concentration found in these patient ocular fluids (500 ng/ml), and cultured for 48 h. Control rat retinal explants were treated with vehicle alone. LDH, a cytosolic enzyme that is released early after cell membranes breakdown into the culture medium, was significantly lower in medium from retinas treated with UDCA at 10 ng/ml and 50 ng/ml, compared to controls (*p* = 0.002 and 0.046, respectively, Mann-Whitney test), but not from those treated at 500 ng/ml (Fig. 2(A)). We used western immunoblotting in order to semi-quantitatively assess cell death pathway marker changes. The necrotic RIP kinase 1 was significantly reduced in retinas treated by UDCA at 10, 50, and 500 ng/ml, as compared with control retinas (*p* = 0.029, Mann-Whitney test) (Fig. 2(B)). The apoptotic protein cleaved-Caspase 3 was significantly reduced in retinas treated by UDCA at 10 ng/ml (*p* = 0.028, Mann-Whitney test) and 50 ng/ml (*p* = 0.014, Mann-Whitney test), but not at 500 ng/ml (Fig. 2(C)). AIF, a Caspase-independent death effector [15], was significantly lower in retinas treated with UDCA at 10, 50, and 500 ng/ml (*p* = 0.029, Mann-Whitney test), compared with control retinas (Fig. 2(D)). One of the early morphological markers in a detached neurosensory retina is the shortening of the outer segments of the photoreceptors. At the lowest concentration of 10 ng/ml, UDCA significantly preserved photoreceptor morphology, as revealed by immunostaining on retinal sections and flat-mounted retinas. Rod outer segment length, labeled by rhodopsin (Rho4D2), was significantly higher in treated retinas, compared with control retinas (*p* = 0.029, Mann-Whitney test) (Fig. 2(E)). In addition, the number of cone photoreceptor segments, specifically labeled on flat-mounted retinas with peanut agglutinin, was significantly higher in UDCA-treated retinas, compared to untreated retinas (*p* = 0.029, Mann-Whitney test) (Fig. 2(F)).

### UDCA, at the Lowest Concentration Measured in Ocular Samples from RD Patients, Was Neuroprotective in Albumin-Exposed Rat Retina Explants

**Fig. 3:**
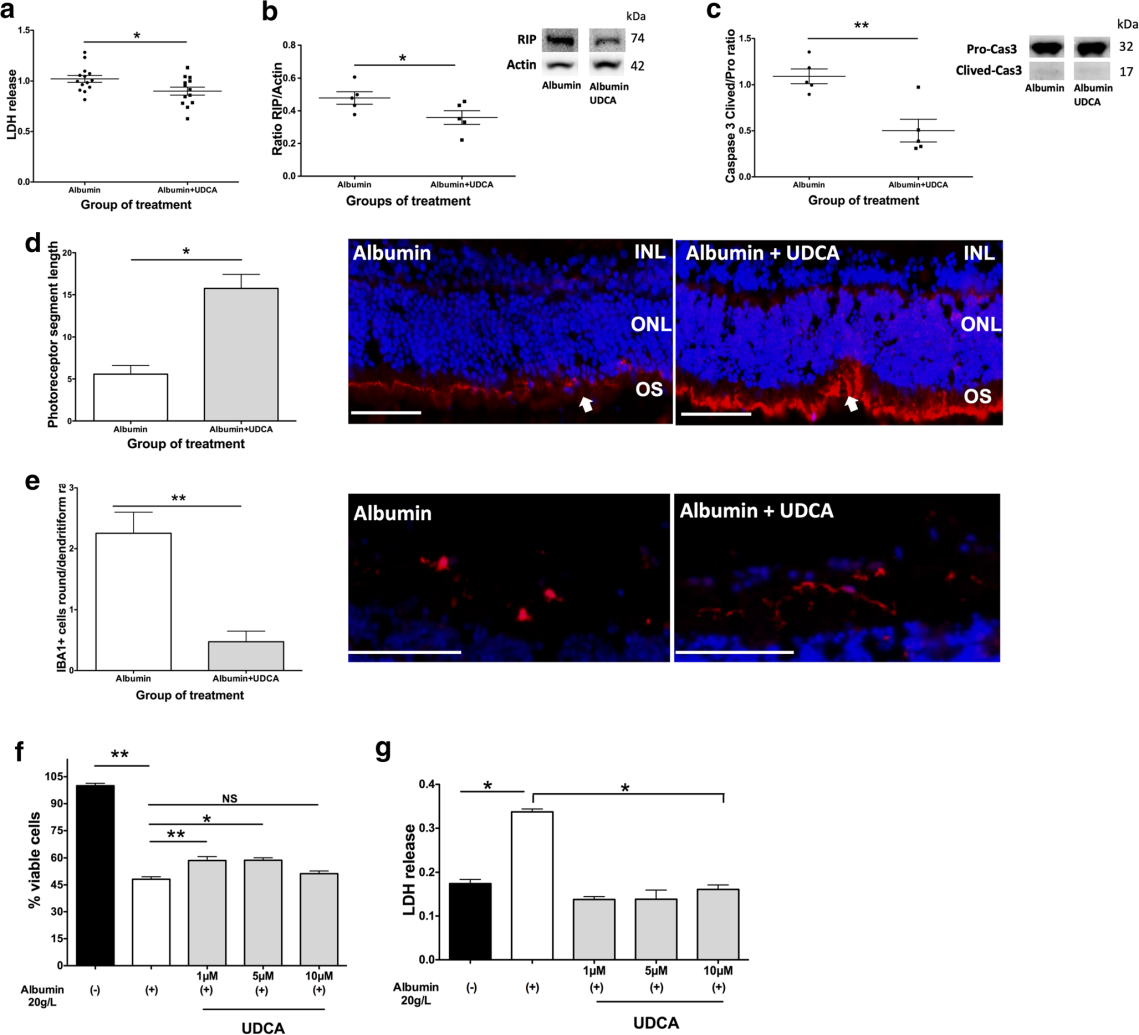
UDCA protects from albumin-induced cell death ex vivo and in vitro. (A–E) Rat retina explants were treated by albumin or albumin and UDCA 10 ng/ml and cultured 6 h. (A) Lactate dehydrogenase (LDH) release was lower in culture medium from retinas treated by UDCA (*p = 0.04). (B–C) Western blotting quantification. Receptor-interacting protein (RIP)/actin ratio was lower in retinas treated by UDCA (*p = 0.02) as well as (C) cleaved/pro-Caspase 3 ratio (**p = 0.008). (D–E) Immunostaining on sections of retinal explants. (D) Rod segments (Rho4D2, red, arrows) length was greater in UDCA-treated retinas (*p = 0.01). Scale: 50 μm. (E) Round/ramified ionized calcium-binding adapter molecule (IBA1)–positive cells (red) ratio was lower in UDCA-treated retinas (**p = 0.008). Scale: 100 μm. (F–G) Human cones were treated by albumin and culture 24 h. UDCA was added 1 h before albumin. (E) The % of viable cells decreased with albumin (**p = 0.001), but was higher when cones received 1 and 5 μM of UDCA (**p = 0.004 and *p = 0.01). (F) Treatment by UDCA (*p = 0.03) decrease LDH release. Mann-Whitney test

As reported above, UDCA concentration in SRF was correlated with protein concentration in SRF (Fig. 1(C)). Thus, rat retinal explants were incubated for 6 h in medium containing albumin at the maximal protein concentration measured in the SRF of RRD patients (12 mg/ml) and compared to control explants treated with medium alone. A significant increase in LDH release was measured in the culture medium of rat neuroretinas exposed to albumin compared to control explants (Supplementary Fig. 3A). The effect of UDCA was then evaluated on rat retinal explants that were exposed to albumin alone (control) or albumin plus UDCA at 10 ng/ml for 6 h. UDCA reduced LDH release significantly (*p* = 0.04, Mann-Whitney test), as compared to albumin exposure alone (Fig. 3(A)). RIP kinase was significantly lower in retinas treated with UDCA (*p* = 0.02, Mann-Whitney test), compared to those exposed to albumin alone (Fig. 3(B)), as well as cleaved-Caspase 3 (*p* = 0.008, Mann-Whitney test) (Fig. 3(C)), demonstrating that markers of both necrosis and apoptosis were significantly reduced in presence of UDCA at the concentration of 10 ng/ml. Additionally, rod segment lengths labeled by Rho4D2 were significantly higher in UDCA-treated retinas compared with untreated retinas (*p* = 0.01, Mann-Whitney test) (Fig. 3(D)). Finally, the number of activated microglia cells (identified as ameboid cells) labeled by IBA1 was significantly reduced by UDCA treatment (*p* = 0.008, Mann-Whitney test) (Fig. 3(E)).

### UDCA Protected WERI-Rb-1 Human Cone-Like Cells from Albumin-Induced Death *In Vitro*

Cone photoreceptor cells are most concentrated in the fovea and are responsible for high acuity vision. Therefore, we tested the effect of albumin on the WERI-Rb-1 human cone-like cell line. Albumin reduced cone viability in a dose-dependent manner (Supplementary Fig. 3B). At the concentration of 20 mg/ml, albumin reduced by 40% cone cell viability after 24 h (Supplementary Fig. 3B). Prior to testing the direct effect of UDCA on WERI-Rb-1 cells, we showed that the bile acid receptors GR, MR, and TGR5 were expressed at the messenger RNA level. Pre-treatment with UDCA at the increasing concentrations of 1, 5, and 10 μM 1 h before albumin exposure significantly increased the number of viable cells at 1 and 5 μM (59% ± 6, mean increase of 11%, *p* = 0.004, and 59% ± 3, mean increase of 11%, *p* = 0.01, respectively, Mann-Whitney test, Fig. 3(F)). LDH release was significantly lower when cone cells had been previously treated with 1, 5, or 10 μM of UDCA (*p* = 0.03 for all concentrations, Mann-Whitney test, Fig. 3(G)).

### Effects of SRF Collected from Patients with RD on Rat Retinal Explants

**Fig. 4:**
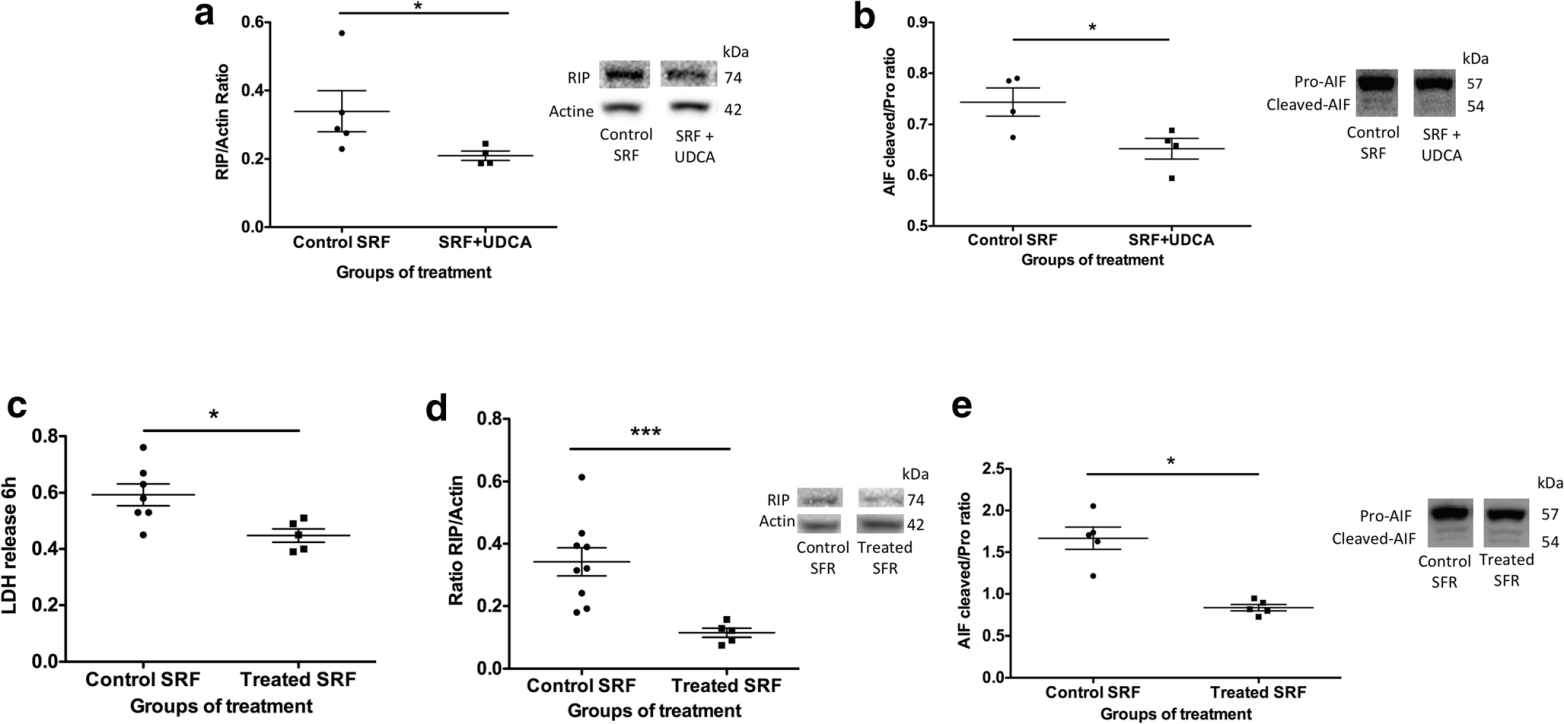
UDCA protects from cell death in subretinal fluid (SRF)–exposed rat retina explants. (A, B) SRF from control patients with and without UDCA (10 ng/ml) was added to rat retina explants for 6 h. (A, B) Western blotting quantification. (A) Receptor-interacting protein (RIP) kinase/actin ratio was significantly lower in retinas treated by SRF + UDCA (*p = 0.03) compared to control SRF. (B) Cleaved /pro-apoptosis-inducing factor (AIF) ratio was significantly lower in retinas treated by SRF + UDCA (*p = 0.03) compared to control SRF. (C–E) SRF from oral UDCA-treated patients and SRF from control patients were added to rat retina explants for 6 h. (C) Lactate dehydrogenase (LDH) release was lower in culture medium from retinas treated by SRF from oral UDCA-treated patients, compared to retinas treated by SRF from control patients (*p = 0.02). (D, E) Western blotting quantification. (D) RIP/actin ratio was lower in retinas treated by SRF from UDCA-treated patients, compared to retinas treated by SRF from control patients (***p = 0.001). (E) Cleaved/pro-AIF ratio was lower in retinas treated by SRF from UDCA-treated patients, compared to retinas treated by SRF from control patients (*p = 0.03). Mann-Whitney test

In order to more accurately mimic clinical conditions, rat retinal explants were incubated with a pool of SRF from RRD patients collected at the time of surgery. SRF from control patients (i.e., those not treated with UDCA) significantly increased markers of necrosis (LDH and RIP 1 kinase), as compared to explants cultured in medium alone (Supplementary Fig. 3 A and C). However, Caspase 3 cleavage was not induced when explants were incubated with SRF. Adding UDCA (10 ng/ml) to SRF from control patients significantly reduced markers of necrosis (RIP, *p* = 0.03, Mann-Whitney test) and Caspase-independent apoptosis (AIF, *p* = 0.03, Mann-Whitney test) (Fig. 4(A, B)). Finally, rat retinal explants were incubated with a pool of SRF collected from UDCA-treated patients in which UDCA levels had been measured. Cell death, quantified by LDH release, was significantly lower when explants were treated with SRF from UDCA-treated patients, as compared to SRF from control patients (*p* = 0.02, Mann-Whitney test) (Fig. 4(C)). RIP kinase and AIF were also significantly lower in retinas incubated with SRF from UDCA-treated patients (*p* = 0.001 and *p* = 0.03, respectively, Mann-Whitney test) (Fig. 4(D, E)).

### Molecular Signature of UDCA in Albumin-Exposed Rat Retinal Explants

**Fig. 5:**
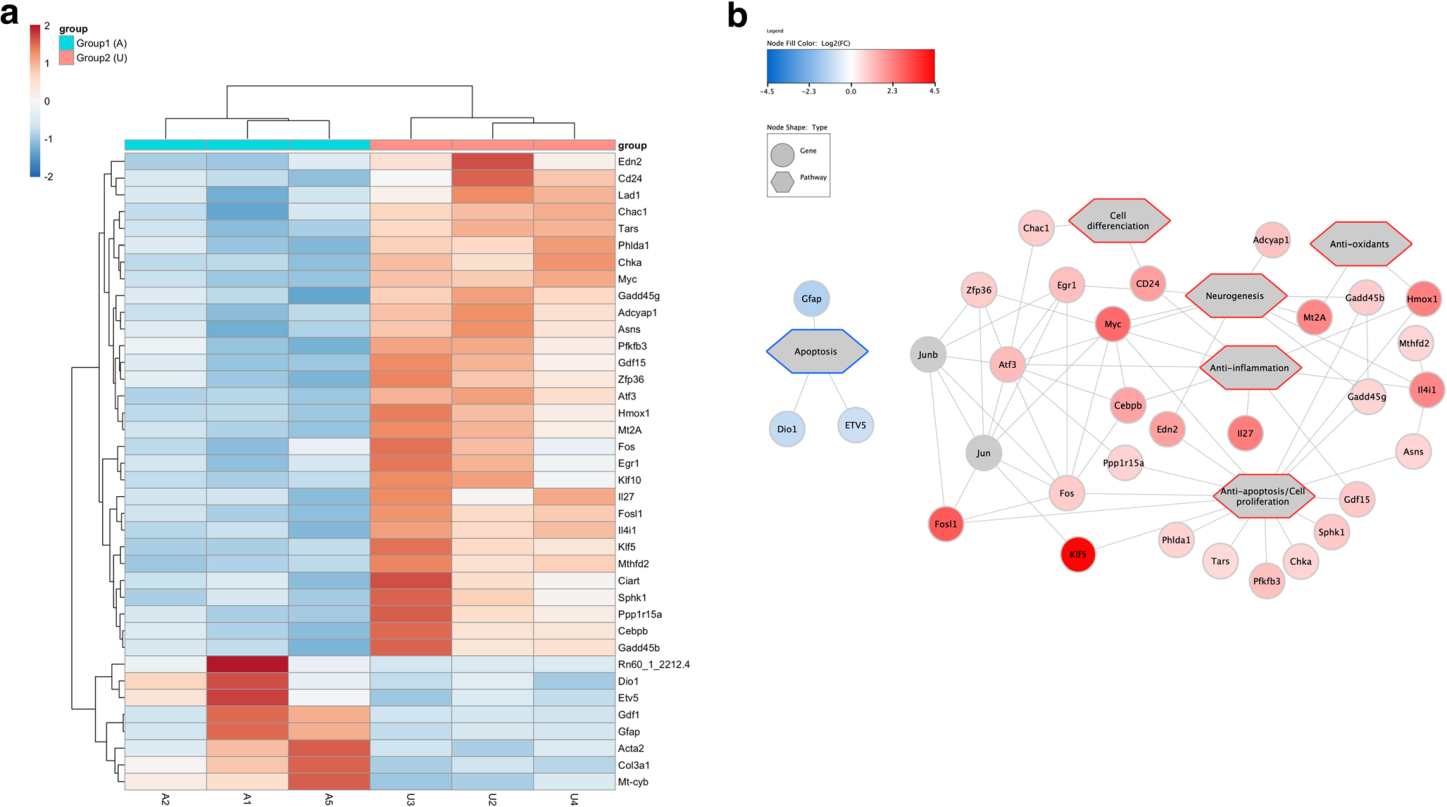
RNA-seq analysis of rat retinal explants exposed to albumin alone or albumin + UDCA over 6 h. (A) Heatmap showed genes upregulated (red) and genes downregulated (blue) by albumin alone (A, group 1) compared to UDCA plus albumin (U, group 2). (B) Network of the main genes upregulated (red) and downregulated (blue) by UDCA and the involved pathways

Transcriptomic differences were analyzed on rat retinal explants exposed for 6 h to albumin at the maximal dose found within the SRF from RD patients (12 mg/ml), and treated or not by UDCA. UDCA treatment significantly altered the expression of 38 genes; 31 were upregulated, and 7 were downregulated (Fig. 5(A)). Enriched analysis showed that hallmark gene sets were mostly implicated in apoptosis regulation, inflammatory response, oxidative stress, and neurogenesis (Fig. 5(B)and Supplementary Table 4), which could mediate the observed biologic effects. Genes upregulated by UDCA, such as *Phlda1*, *Gdf15*, *Tars*, *Asns*, *Pfkfb3*, Chka, CD24, *Hmox1*, and *Sphk1*encode proteins, involved in cell survival pathways, while *Adcyap1*, *Atf3*, *Mt2A*, *Il4i1*, and *Egr1 and 2* encode proteins promoting neuroregeneration. *Myc*, *Edn2*, *Gadd45b*, and *Gadd45g* encode proteins involved in both survival pathways in the retina and neuroregeneration. *Hmox1 and Mt2A*, encoding proteins with anti-oxidant properties, were also upregulated. On the other hand, UDCA also upregulated genes that were implicated in inflammatory response regulation, such as *Il27*, Il4i1, *Atf3*, *Cebpb*, *Gdf15*, *Zfp36*, *Hmox1*, and Nr4A1.

## Discussion

UDCA, the first-line therapy for cholestatic liver diseases [11], has shown beneficial neuroprotective effects in various animal models of retinal diseases [6], but the ocular bioavailability of oral UDCA has not been previously evaluated, therefore limiting its clinical use. In this translational study, we demonstrated that in patients with retinal detachment, oral UDCA reaches effective concentrations in the SRF. UDCA ocular bioavailability positively correlated with the surface area of the RRD and with the protein concentration in the SRF, hence suggesting that blood retinal barrier breakdown is the main factor that influences the ocular bioavailability of UDCA administered orally at 10 mg/kg/day. Surgical factors that alter the blood retinal barrier integrity, such as cryotherapy, could have influenced UDCA intraocular levels. However, in this study, all samples were taken before cryotherapy or any laser has been performed, excluding any interference with ocular biodisponibility of UDCA. These findings are consistent with observations in patients with amyotrophic lateral sclerosis [5, 12], a condition in which the blood-brain barrier is compromised [16]. In these patients, oral UDCA administered at doses ranging from 15 to 50 mg/kg/day crossed the blood-brain barrier in a dose-dependent manner. At the dosage of 10 mg/kg/day, the tolerance and safety of oral UDCA was confirmed in our study with only mild gastrointestinal tract adverse effects, as previously reported [12]. Finally, incubation with the SRF collected from patients and who had received oral UDCA reduced cell death in rat retinal explants, thereby suggesting that efficacious UDCA levels were reached in patients after oral administration. The correlation between visual outcome and ocular UDCA levels was not significant in this study that was not designed nor powered to show it. In addition, patients with higher UDCA levels were those with more extended RRD, and thus those expected to have worse visual outcome. But encouragingly, the later patients did not have worse visual outcome in this study. Only a randomized controlled study with clinically homogenous RRD patients could determine the functional and anatomical benefit of UDCA in patients with RRD. Whether UDCA reaches the eye mainly through the outer blood retinal barrier remains to be elucidated. At similar time duration between UDCA administration and ocular media sampling, UDCA was detected in vitreous, in SRF, or in both ocular fluids, while retinal levels could not be determined. UDCA concentration ranges in vitreous and SRF were similar which indicates the very low probability of infusion contamination. UDCA was measured in both compartments in 4 eyes and in one compartment in 8 eyes suggesting that low vitreous contamination occurred during SRF sampling. UDCA was measured in both compartments in 4 eyes and in one of the two compartments in 8 eyes suggesting that low vitreous contamination occurred during SRF sampling.

We thus believe that contamination of the LSR with vitreous or infusion fluid is very unlikely because the sampling was performed prior to any vitrectomy, limiting this risk as much as technically possible. But, if the SRF had been diluted with infusion fluid, we may have slightly underestimated the levels of UDCA in the SRF and may not have detected UDCA in some SRF. This would have led us to rather underestimate the passage of UDCA and therefore supports our results showing that oral administration could be used for patients with RD. In addition, in UDCA, being a hydrophobic compound, it is expected that retinal UDCA levels could be higher than ocular fluid ones. The passage of UDCA in the eye is probably controlled by many parameters, such as transporters, efflux proteins, and the integrity of the BRB. The fact that in untreated controls patients UDCA was measured in one single vitreous suggests that interindividual variability also controls the passage of endogenous UDCA into the eye.

Several approaches were implemented in exploring the possible benefit of UDCA and its mechanisms of action on cell death induced by retinal detachment. Incubation with UDCA at 10 ng/ml (a concentration measured in SRF of patients) protected retina explants from necrosis and caspase-dependent and -independent cell death, as shown by the decreases of LDH release, RIP kinase activation, Caspase 3 cleavage, and AIF levels. At lower doses, UDCA also preserved the integrity of photoreceptor outer segments, which is recognized as a major visual prognosis factor in patients with RD [4]. The reason why higher doses of UDCA were not protective could potentially be related to the high hydrophobicity of the molecule, inducing direct interactions with the cell membrane, independently from receptor-mediated effects. Similarly, we also showed that glucocorticoids can exert toxic effects at higher doses, when in direct contact with the retina [17].

Various molecular components of SRF could exert toxicity on retinal neurons [13, 18] that, in physiologic conditions, are maintained by blood retinal barriers in a confined and controlled microenvironment. With increased RRD duration, SRF protein concentration increases [19] and visual prognosis worsens [20], but the direct effect of proteins on photoreceptors has not yet been evaluated. In the present study, protein concentration in SRF correlated with the detachment surface area. In addition, we showed that albumin reduced the viability of cultured immortalized human cone photoreceptors, suggesting a direct, toxic effect of albumin. Similarly, Liu et al. showed that cortical neurons are damaged by albumin uptake [21]. By adding albumin at the concentration measured in SRF from patients to retinal explants, in which the neuroretina is separated from the RPE, we intended to create an ex vivo RD model closer to the human clinical disease, in order to evaluate the neuroprotective properties of bile acids. Even in the presence of albumin, UDCA was still effective in reducing apoptosis and necrosis markers, as well as protecting photoreceptor integrity. However, the rat model has some limitations such as the absence of pigmented RPE and no macula. These results could thus be confirmed in retinal explants from other rodent strains, particularly pigmented ones as well as in human retina, although recent work showed that mechanisms of cell death in human retina explants are similar to those occurring in rat explants [22]. Since availability of human retinas is limited according to countries legislations, we tried to overcome the absence of fovea by additionally showing UDCA protection on human cone-like cells.

The protective effect of TUDCA was previously demonstrated against caspase activation in an *in vivo* RD model [7] and several models of photoreceptor degeneration [8], as well as against non-caspase-dependent mechanisms involving AIF in retinal cells exposed to glucose [23]. The exact contribution of taurine, the most abundant and neuroprotective amino acid in the retina [24] in these effects, had not been previously evaluated. Here, we showed that UDCA, which does not contain taurine, protected from apoptosis, and also from necrosis. When rat retinal explants were exposed to SRF from patients who had not received UDCA, caspase-dependent cell death activation was not observed, suggesting that SRF could have anti-apoptotic proprieties, and that caspase-dependent apoptosis may not occur in human RRD. In line with this hypothesis, although enzymatic activities of caspases have been reported in experimental models of RD [7], Caspase inhibition by pan-Caspase inhibitors failed to prevent photoreceptor loss [2].

Bile acid receptors have been described in the liver, intestine, and brain, and include nuclear receptors (Farnesoid X receptor (FXR), vitamin D receptor, pregnane X receptor, GR, and MR) and membrane receptors (TGR5, sphingosine 1-phosphate receptor 2, and α5β1 Integrin) [6]. Whether the beneficial effects of UDCA observed in the retina were mediated by these receptors is beyond the scope of this study. However, all of these receptors, with the exception of FXR, have been identified in the retina, and specific interactions have been reported for TUDCA and TGR5 in retinal ganglion cells [25].

UDCA altered the transcriptomic regulation of genes encoding proteins with anti-oxidant activities. *Hmox1*, which encodes heme oxygenase-1, was significantly upregulated by UDCA, and is known to exert neuroprotection in the retina against oxidative stress injury [26], optical nerve section [27], and diabetic conditions [28]. In addition, the overexpression of heme oxygenase-1 in a mouse model of RD protected photoreceptors from apoptosis [29]. *Mt2A*, which encodes metallothionein 2A protein, protecting against hydroxyl free radicals and metal iron toxicity, preserved from various forms of retinal degeneration [30]. *Atf3*, upregulated by UDCA, is a member of the cAMP-response element binding protein family, which was upregulated in models of retinal and optic nerve regeneration [31], and a mutant *Atf3* mouse which showed reduced nerve regeneration [32]. Other genes upregulated by UDCA also encode proteins with potential neuroprotectant effects. *Adcyap1*, which encodes pituitary adenylate cyclase-activating polypeptide (PACAP), was shown to carry neuroprotective properties in retinal degeneration and optic nerve crush models [33, 34]. Endothelin 2 showed protection of mutant photoreceptors in inherited photoreceptor degeneration [35], as well as protection against light damage [36]. *Cd24* is a cell surface receptor expressed in photoreceptor precursors [37] and *Chac1*, which encodes a member of the gamma-glutamylcyclotransferase family of proteins, shown to promote neuronal differentiation and regulate glutathione levels [38]. Many genes involved in cell death were regulated, but more importantly, genes encoding anti-apoptotic proteins, such as *Tars*, *Myc*, *Adcyap1*, *Asns*, *Hmox1*, and *Pfkfb3*, and the anti-necrotic protein *Hmox1*, were upregulated, identifying some of the potential mechanisms of action of UDCA. Additionally, *Il27*, which was upregulated, promotes the expression of the anti-oxidative protein complement factor H, and reduces inflammation through IL10 expression in the retina [39, 40]. Through the release of pro-inflammatory cytokines [14], activated microglia are suspected to be neurotoxic to photoreceptor cells. Here, UDCA reduced the number of activated (ameboid) compared with normal (ramified) microglia, and we hypothesized that the observed normal microglia shape in UDCA-treated explants could be explained by the anti-inflammatory effects of UDCA. Altogether, these results indicate that, through transcriptional effects, UDCA regulates not only cell death pathways, but also retinal regeneration and inflammatory pathways.

In conclusion, this pilot study indicates that UDCA reaches the SRF proportionally to the extent of blood retinal barrier rupture. It reaches neuroprotective concentrations that efficiently reduce apoptosis and necrosis and preserve photoreceptor integrity. UDCA induces the expression of retinal genes with anti-oxidant, anti-apoptotic, and anti-inflammatory properties. These results suggest that oral UDCA should be further evaluated clinically as adjuvant treatment to surgery in RD patients.

## Supplementary Information

Supplementary Fig. 1 Since Ursodeoxycholic acid (UDCA) is hydrophobic, the first dilution (10 mg/ml) was always performed in ethanol 100%. The second dilution (1: 25) was performed in PBS and the third dilution (1:20) on medium (UDCA 20 μg/ml, ethanol 0.2%). Then, to reach the 3 different final concentrations of UDCA in the entire volume of the well (500, 50 and 10 ng/ml) subsequent dilutions were performed with medium. The final dilution of 1:40 corresponded to the final dilution on the culture well. When albumin was used in the experiments, it was added at the last dilution before addition into culture wells to obtain an albumin concentration of 12 mg/ml and UDCA final concentration of 10 ng/ml. When subretinal fluid (SRF) was used in the experiments, all dilution steps (including the first step with ethanol) were followed but the two last dilutions before addition into culture wells were performed in SRF and not in medium. Vehicle solutions without UDCA followed the same dilutions steps (including the first step with ethanol). (PNG 382 kb)

High Resolution Image (TIFF 792 kb)

Supplementary Fig. 2 UDCA concentrations in ocular fluids and visual recovery after surgery for retinal detachment (RD). Best-corrected visual acuity (BCVA) change at month-6 after surgery as a function of UDCA concentrations in SRF (A), vitreous (B) and plasma (C) (Spearman correlation, *P* = 0.7, 0.7 and 0.4, respectively) (PNG 177 kb)

High Resolution Image (TIFF 619 kb)

Supplementary Fig. 3 Albumin and subretinal fluid (SRF) induced cell death (A) Lactate dehydrogenase (LDH) release was significantly lower in culture medium from control retinas, compared to albumin-treated retinas (*p = 0.01, Mann-Whitney test) and SRF-treated retinas (*p = 0.04, Mann-Whitney test). **(B)** Human cones (WERI-Rb-1 cell line) were treated at progressive concentrations by albumin (from 2.5 to 40 g/L) during 24 h. Cell Titer was assessed in order to determine the % of viable cells. Albumin concentrations higher than 5 g/L significantly reduced viability of cells, when compared to the control (*p < 0.05, Mann-Whitney test). A concentration of albumin at 20 g/L induced a mean of 40% of cell viability reduction. **(C)** Western blotting and quantitative analysis were performed for receptor-interacting protein (RIP) kinase. RIP kinase reported on Actin was significantly lower in non-cultured retinas (NC Control), compared to medium (Control) or SRF-treated retinas (**p = 0.008, respectively, Mann-Whitney test). SRF did not induce the cleaved form of Caspase 3. (PNG 218 kb)

High Resolution Image (TIFF 692 kb)

Table S1 (DOCX 21 kb)

Table S2 (DOCX 13 kb)

Table S3 (DOCX 17 kb)

Table S4 (XLSX 19 kb)

ESM 1 (PDF 308 kb)

## Declarations

### Disclaimer

The sponsors did not have any role in the study.
