# Supplementary figures and images for: Oral Ursodeoxycholic Acid Crosses the Blood Retinal Barrier in Patients with Retinal Detachment and Protects Against Retinal Degeneration in an Ex Vivo Model
Source: Neurotherapeutics. 2021 Feb 3;18(2):1325–38. doi: 10.1007/s13311-021-01009-6 (PMC8423962; doi:10.1007/s13311-021-01009-6)

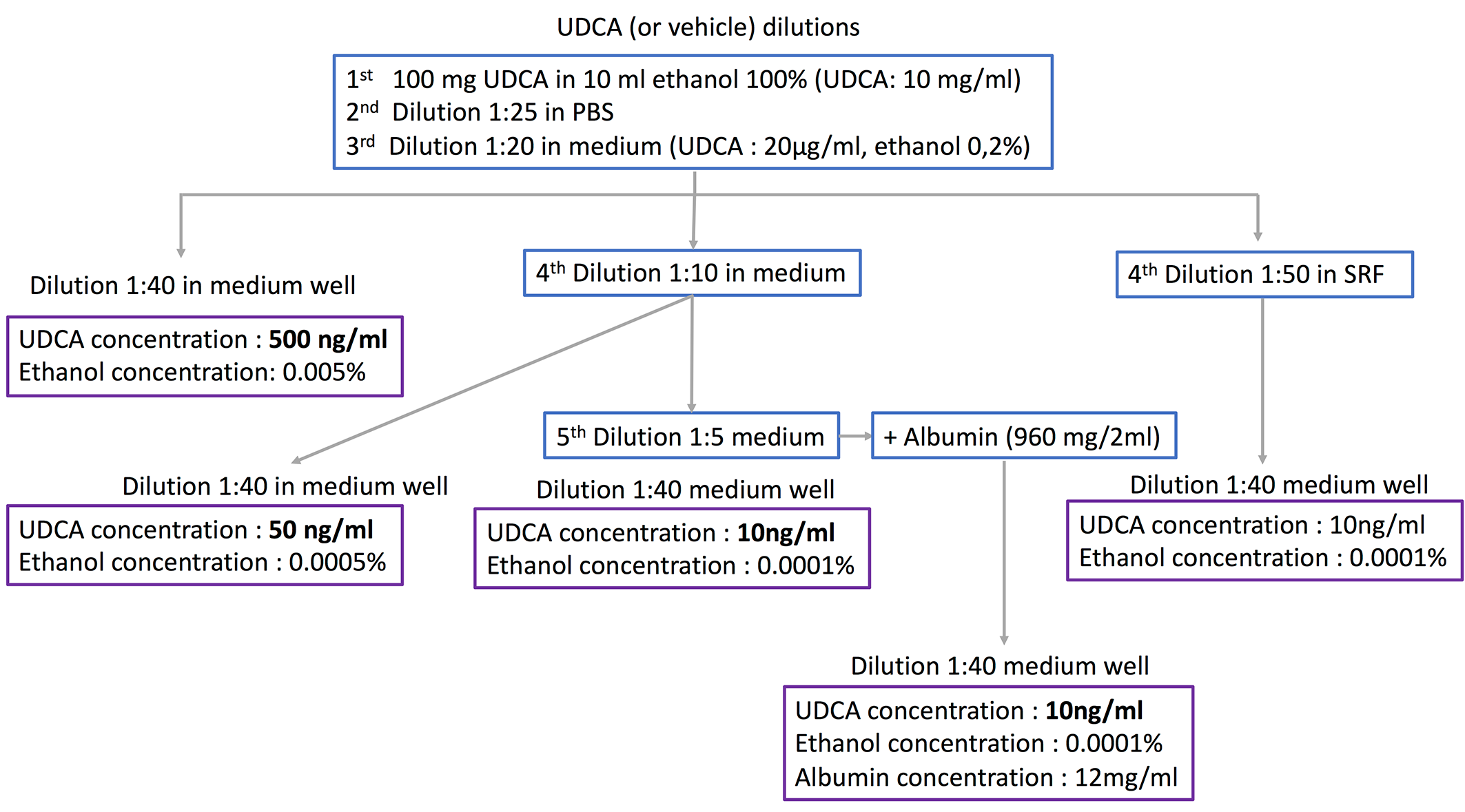

Supplement: Supplementary file 1 — Since Ursodeoxycholic acid (UDCA) is hydrophobic, the first dilution (10 mg/ml) was always performed in ethanol 100%. The second dilution (1: 25) was performed in PBS and the third dilution (1:20) on medium (UDCA 20 μg/ml, ethanol 0.2%). Then, to reach the 3 different final concentrations of UDCA in the entire volume of the well (500, 50 and 10 ng/ml) subsequent dilutions were performed with medium. The final dilution of 1:40 corresponded to the final dilution on the culture well. When albumin was used in the experiments, it was added at the last dilution before addition into culture wells to obtain an albumin concentration of 12 mg/ml and UDCA final concentration of 10 ng/ml. When subretinal fluid (SRF) was used in the experiments, all dilution steps (including the first step with ethanol) were followed but the two last dilutions before addition into culture wells were performed in SRF and not in medium. Vehicle solutions without UDCA followed the same dilutions steps (including the first step with ethanol). (PNG 382 kb) [file 13311_2021_1009_Fig6_ESM.png]

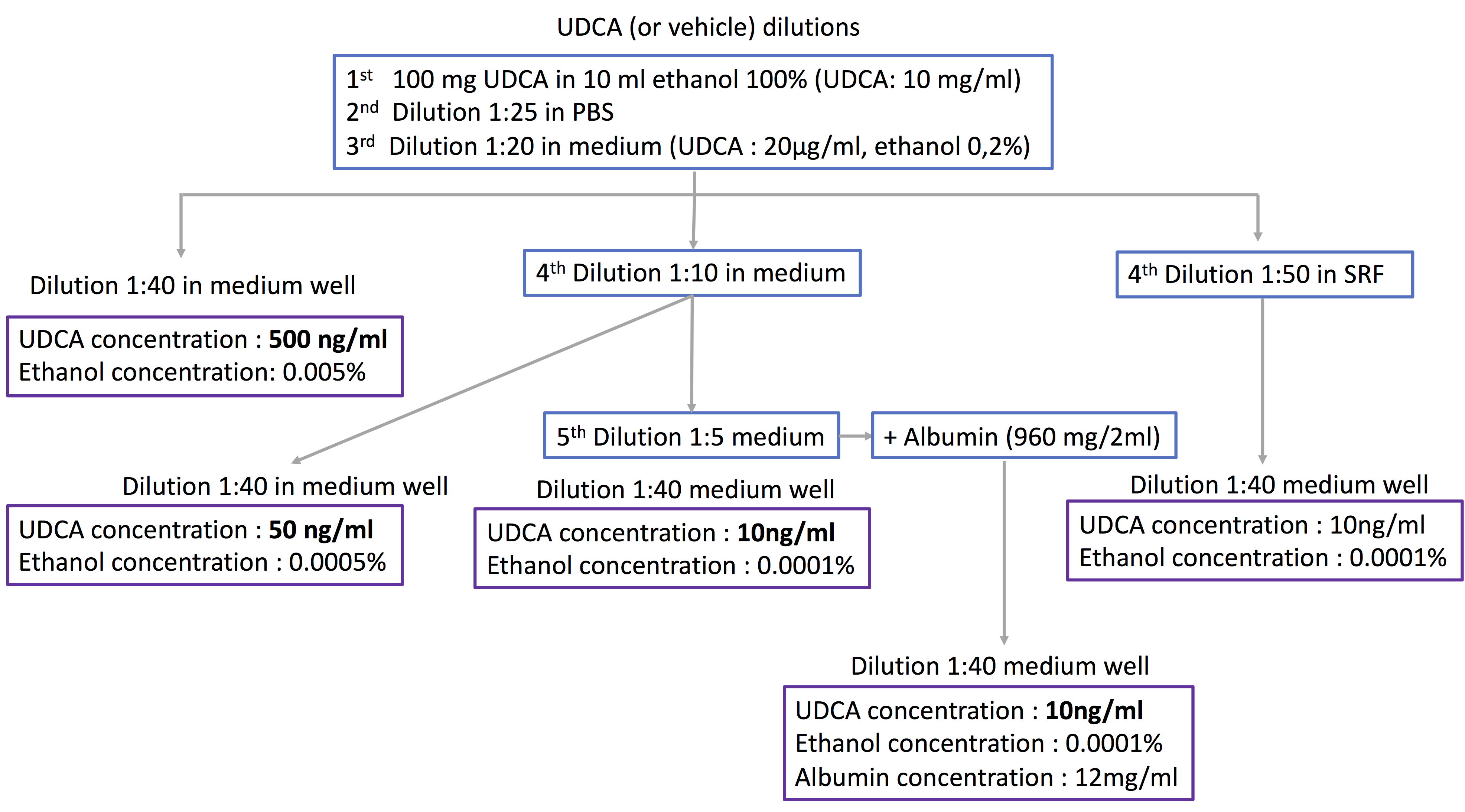

Supplement: Supplementary file 2 — High Resolution Image (TIFF 792 kb) [file 13311_2021_1009_MOESM1_ESM.tiff]

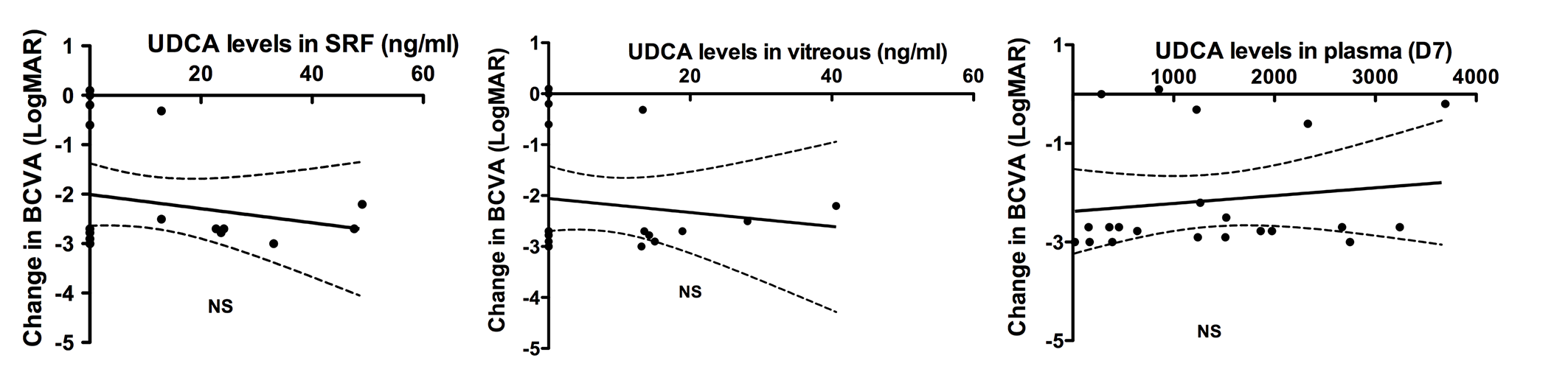

Supplement: Supplementary file 3 — UDCA concentrations in ocular fluids and visual recovery after surgery for retinal detachment (RD). Best-corrected visual acuity (BCVA) change at month-6 after surgery as a function of UDCA concentrations in SRF (A), vitreous (B) and plasma (C) (Spearman correlation, P = 0.7, 0.7 and 0.4, respectively) (PNG 177 kb) [file 13311_2021_1009_Fig7_ESM.png]

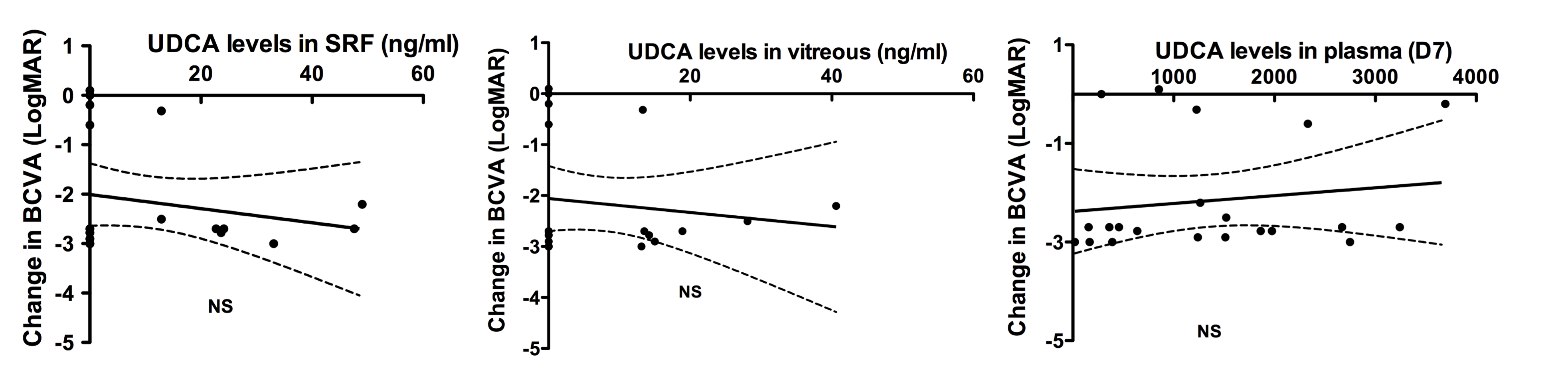

Supplement: Supplementary file 4 — High Resolution Image (TIFF 619 kb) [file 13311_2021_1009_MOESM2_ESM.tiff]

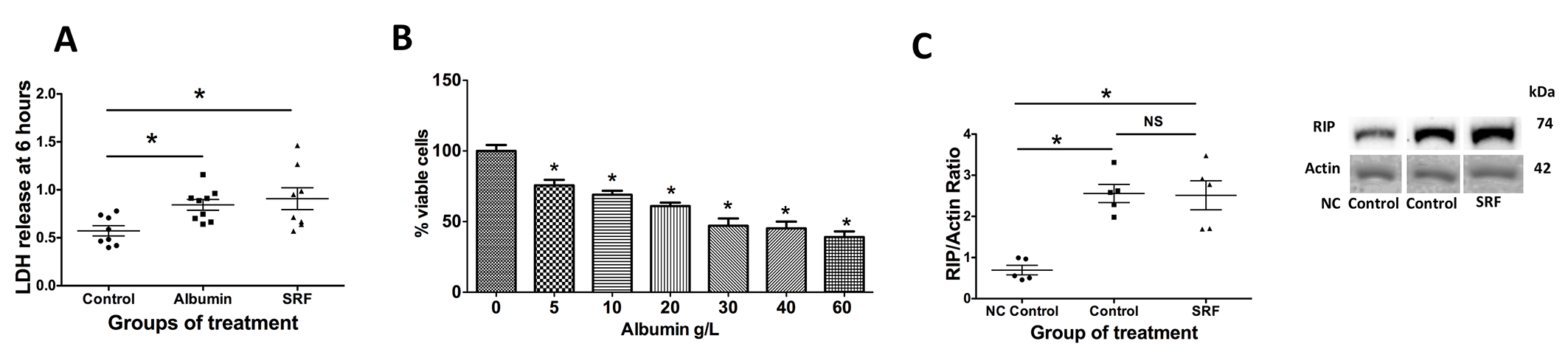

Supplement: Supplementary file 5 — Albumin and subretinal fluid (SRF) induced cell death (A) Lactate dehydrogenase (LDH) release was significantly lower in culture medium from control retinas, compared to albumin-treated retinas (*p = 0.01, Mann-Whitney test) and SRF-treated retinas (*p = 0.04, Mann-Whitney test). (B) Human cones (WERI-Rb-1 cell line) were treated at progressive concentrations by albumin (from 2.5 to 40 g/L) during 24 h. Cell Titer was assessed in order to determine the % of viable cells. Albumin concentrations higher than 5 g/L significantly reduced viability of cells, when compared to the control (*p < 0.05, Mann-Whitney test). A concentration of albumin at 20 g/L induced a mean of 40% of cell viability reduction. (C) Western blotting and quantitative analysis were performed for receptor-interacting protein (RIP) kinase. RIP kinase reported on Actin was significantly lower in non-cultured retinas (NC Control), compared to medium (Control) or SRF-treated retinas (**p = 0.008, respectively, Mann-Whitney test). SRF did not induce the cleaved form of Caspase 3. (PNG 218 kb) [file 13311_2021_1009_Fig8_ESM.png]

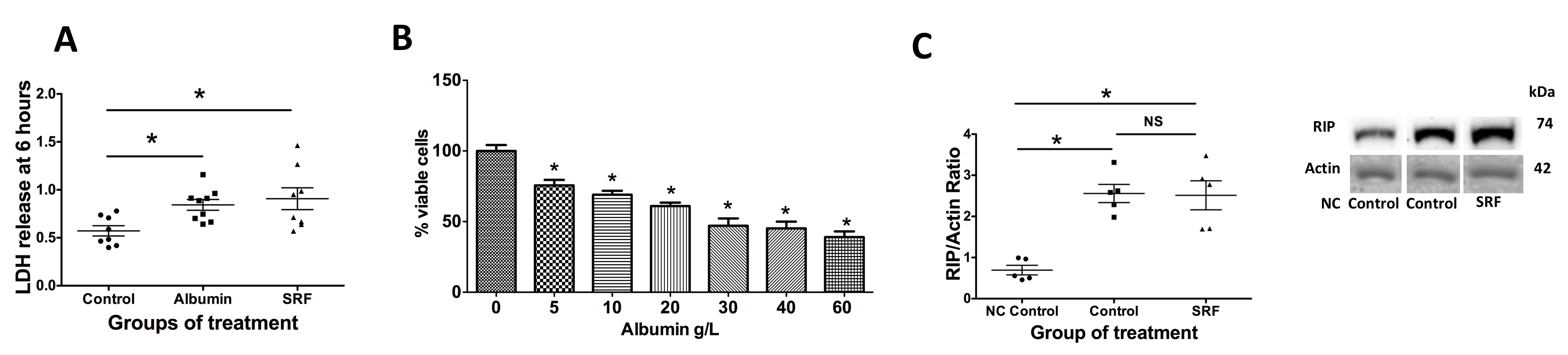

Supplement: Supplementary file 6 — High Resolution Image (TIFF 692 kb) [file 13311_2021_1009_MOESM3_ESM.tiff]
