# Supplementary material for: Oral Ursodeoxycholic Acid Crosses the Blood Retinal Barrier in Patients with Retinal Detachment and Protects Against Retinal Degeneration in an Ex Vivo Model
Source: Neurotherapeutics. 2021 Feb 3;18(2):1325–38. doi: 10.1007/s13311-021-01009-6 (PMC8423962; doi:10.1007/s13311-021-01009-6)
Supplement: Supplementary file 7 — (DOCX 21 kb) [file 13311_2021_1009_MOESM4_ESM.docx]

**Supplementary Table 1**. Clinical characteristics and UDCA levels in plasma and ocular fluids in treated and controls patients with rhegmatogenous retinal detachment

| Patient | Age | Sex | Baseline BCVA (20ft) | RD type | RD  Extension (clock hours) | Macula status | Lens status | UDCA level in plasma before treatment (ng/ml) | UDCA intake before surgery  (hours) | Type of Surgery | UDCA level in SRF (ng/ml) | UDCA level in vitreous (ng/ml) | Protein level in SRF | UDCA level in plasma at day 7 after treatment (ng/ml) | Month 6 BCVA  (20ft) |
| --- | --- | --- | --- | --- | --- | --- | --- | --- | --- | --- | --- | --- | --- | --- | --- |
| 1 | 51 | M | HM | simple | 5 | off | phakic | 13.1 | 6-8 | VCG | 0 | 0 | 1.62 | 1975.1 | 20/32 |
| 2 | 68 | M | HM | simple | 5.5 | off | pseudophakic | 30.8 | 6-8 | VCG | 0 | 0 | 1.27 | 390.6 | 20/20 |
| 3 | 49 | M | HM | simple | 3.5 | off | pseudophakic | 0 | ≥ 12 | VCG | 0 | 0 | 1.31 | 167.3 | 20/20 |
| 4 | 80 | F | 20/100 | simple | 3.5 | off | pseudophakic | 13.3 | 9-11 | VCG | 0 | 0 | 2.63 | 2329.6 | 20/25 |
| 5 | 68 | F | HM | simple | 4.5 | off | pseudophakic | 0 | ≥ 12 | VCG | 0 | 0 | 1.91 | 156.9* | 20/40 |
| 6 | 46 | M | 20/50 | simple | 3.5 | off | pseudophakic | 12.8 | 6-8 | VCG | 0 | 0 | 1.91 | 3694.2 | 20/32 |
| 7 | 53 | F | HM | simple | 4.5 | off | phakic | 0 | 9-11 | VLS | 0 | 0 | 1.24 | 1239.2 | 20/25 |
| 8 | 48 | M | 20/20 | simple | 2 | on | phakic | 0 | 9-11 | VCG | 0 | 0 | 0.59 | 232.2 | 20/20 |
| 9 | 54 | M | 20/20 | simple | 3 | on | phakic | 0 | 6-8 | VCG | 0 | 0 | 1.03 | 854 | 20/25 |
| 10 | 55 | F | HM | simple | 4 | off | phakic | 0 | 6-8 | VCG | 0 | 15 | 1.62 | 1513.4 | 20/25 |
| 11 | 60 | M | HM | simple | 5 | off | pseudophakic | 0 | ≤ 5 | VCG | 0 | 13.1 | 2.02 | 19.5* | 20/20 |
| 12 | 56 | F | HM | simple | 3.5 | off | phakic | 0 | ≤ 5 | VCG | 0 | 14.2 | 1.58 | 1862.3 | 20/32 |
| 13 | 80 | F | HM | simple | 4.5 | off | pseudophakic | 0 | 6-8 | VCG | 0 | 13.5 | 1.52 | 3242.9 | 20/40 |
| 14 | 52 | M | HM | simple | 3 | off | pseudophakic | 14.7 | ≥ 12 | VCG | 33.1 | 0 | 3.92 | 2748.3 | 20/20 |
| 15 | 64 | M | HM | simple | 6 | off | phakic | 13.2 | ≤ 5 | VCG | 23.6 | 0 | 7.17 | 638.4 | 20/32 |
| 16 | 72 | M | HM | simple | 6 | off | phakic | 58.3 | ≤ 5 | VCG | 24.1 | 0 | 4.98 | 457.1* | 20/40 |
| 17 | 64 | M | HM | simple | 6 | off | phakic | 0 | ≤ 5 | VCG | 47.6 | 0 | 5.70 | 362.3 | 20/40 |
| 18 | 79 | F | HM | simple | 7.5 | off | pseudophakic | 54.8 | ≥ 12 | VCS | 22.7 | 18.9 | 5.49 | 2670 | 20/40 |
| 19 | 35 | M | HM | complex | 12 | off | phakic | 0 | ≥ 12 | VCPG | 49 | 40.6 | 10.47 | 1262.9 | 20/125 |
| 20 | 67 | M | HM | complex | 12 | off | pseudophakic | 29.8 | 9-11 | VCPS | 12.9 | 28.1 | 2.35 | 1522 | 20/63 |
| 21 | 57 | M | 20/63 | simple | 3 | on | pseudophakic | 66.6 | ≤ 5 | VCG | 12.9 | 13.3 | 3.27 | 1226.8 | 20/32 |
| 22 | 65 | F | 20/32 | simple | 2.5 | on | phakic | 0 | control | VCG | 0 | 13.1 | control | 0 | 20/25 |
| 23 | 80 | M | HM | complex | 11 | off | pseudophakic | 0 | control | VCPS | 0 | 0 | control | 0 | 20/50 |
| 24 | 69 | M | 20/32 | complex | 3 | on | phakic | 15 | control | VCPG | 0 | 0 | control | 62.4 | 20/40 |
| 25 | 54 | F | 20/20 | simple | 3 | on | phakic | 0 | control | VCLS | 0 | 0 | control | 0 | 20/25 |
| 26 | 69 | M | 20/40 | simple | 2.5 | on | pseudophakic | 0 | control |  | 0 | 0 | control | 14.3 | 20/25 |

SFR : subretinal fluid ; BCVA : Best-corrected visual acuity ; HM: hand motion, M : Male ; F : Female,* Patients that stopped treatment before Day 7, V : vitrectomy, C : Cryotherapy, G :Gaz tamponade, L : Photocoagulation Laser, S : Silicone tamponade, P: peeling
