# Supplementary material for: Oral Ursodeoxycholic Acid Crosses the Blood Retinal Barrier in Patients with Retinal Detachment and Protects Against Retinal Degeneration in an Ex Vivo Model
Source: Neurotherapeutics. 2021 Feb 3;18(2):1325–38. doi: 10.1007/s13311-021-01009-6 (PMC8423962; doi:10.1007/s13311-021-01009-6)
Supplement: Supplementary file 8 — (DOCX 13 kb) [file 13311_2021_1009_MOESM5_ESM.docx]

**Supplemental Table 2**: Patient-reported drug adverse effects questionnaire

| Did you have stomach pain? | YES | NO |
| --- | --- | --- |
| Did you have diarrhea? | YES | NO |
| Have you had any episodes of constipation? | YES | NO |
| Have you felt heavy digestion? | YES | NO |
| Did you emit excessive digestive gas (flatulencies)? | YES | NO |
| Have you had a rash (pimples)? | YES | NO |
| Did you feel unwell? | YES | NO |
| Did you have difficulty breathing? | YES | NO |
| Did you have an unpleasant taste when taking the medicine or right after? | YES | NO |
| Have you had fever or chills? | YES | NO |
| Did you have a headache? | YES | NO |
| Did you have an episode of blurred vision (in the unoperated eye)? | YES | NO |
| Did you feel tingling in your arms or legs? | YES | NO |
