# Supplementary material for: Oral Ursodeoxycholic Acid Crosses the Blood Retinal Barrier in Patients with Retinal Detachment and Protects Against Retinal Degeneration in an Ex Vivo Model
Source: Neurotherapeutics. 2021 Feb 3;18(2):1325–38. doi: 10.1007/s13311-021-01009-6 (PMC8423962; doi:10.1007/s13311-021-01009-6)
Supplement: Supplementary file 9 — (DOCX 17 kb) [file 13311_2021_1009_MOESM6_ESM.docx]

**Supplemental Table 3**: Reported side-effects in patients treated with ursodeoxycholic acid and controls during 6 months follow-up

| **Side effects** | **Day 1** | **Day 7** | **Month 1** | **Month 2** | **Month 3** | **Month 4** | **Month 6** | **Total*** |
| --- | --- | --- | --- | --- | --- | --- | --- | --- |
| **Nausea** | TG=0  CG=0 | TG=1  CG=0 | TG=0  CG=0 | TG=0  CG=0 | TG=0  CG=0 | TG=0  CG=0 | TG=0  CG=0 | TG=1  CG=0 |
| **Epigastric pain** | TG=0  CG=0 | TG=3  CG=0 | TG=1  CG=0 | TG=1  CG=0 | TG=0  CG=0 | TG=2  CG=0 | TG=0  CG=0 | TG=7  CG=0 |
| **Flatulence** | TG=0  CG=0 | TG=7  CG=1 | TG=2  CG=0 | TG=2  CG=0 | TG=1  CG=0 | TG=2  CG=0 | TG=0  CG=0 | TG=14  CG=1 |
| **Diarrhea** | TG=1  CG=0 | TG=0  CG=0 | TG=3  CG=0 | TG=1  CG=0 | TG=1  CG=0 | TG=1  CG=0 | TG=1  CG=0 | TG=8  CG=0 |
| **Constipation** | TG=0  CG=0 | TG=3  CG=0 | TG=6  CG=0 | TG=2  CG=0 | TG=0  CG=0 | TG=1  CG=0 | TG=0  CG=0 | TG=12  CG=0 |
| **Unpleasant taste** | TG=0  CG=0 | TG=3  CG=0 | TG=3  CG=0 | TG=2  CG=0 | TG=2  CG=0 | TG=1  CG=0 | TG=0  CG=0 | TG=11  CG=0 |
| **Heavy digestion** | TG=0  CG=0 | TG=2  CG=0 | TG=0  CG=0 | TG=0  CG=0 | TG=0  CG=0 | TG=0  CG=0 | TG=0  CG=0 | TG=2  CG=0 |
| **Tingling** | TG=0  CG=0 | TG=2  CG=0 | TG=2  CG=1 | TG=2  CG=0 | TG=1  CG=0 | TG=0  CG=0 | TG=1  CG=0 | TG=8  CG=1 |
| **Trouble vision fellow eye** | TG=0  CG=1 | TG=5  CG=1 | TG=1  CG=0 | TG=2  CG=0 | TG=0  CG=0 | TG=0  CG=0 | TG=0  CG=0 | TG=8  CG=2 |
| **Fatigue** | TG=0  CG=0 | TG=0  CG=0 | TG=2  CG=0 | TG=0  CG=0 | TG=1  CG=0 | TG=0  CG=0 | TG=0  CG=0 | TG=3  CG=0 |
| **Headache** | TG=1  CG=1 | TG=4  CG=2 | TG=4  CG=2 | TG=2  CG=0 | TG=0  CG=0 | TG=2  CG=0 | TG=0  CG=0 | TG=13  CG=5 |
| **Discomfort** | TG=0  CG=0 | TG=1  CG=0 | TG=0  CG=0 | TG=0  CG=0 | TG=0  CG=0 | TG=0  CG=0 | TG=0  CG=0 | TG=1  CG=0 |
| **Skin rash** | TG=0  CG=0 | TG=0  CG=0 | TG=0  CG=0 | TG=1  CG=0 | TG=0  CG=0 | TG=0  CG=0 | TG=0  CG=0 | TG=1  CG=0 |

TG=treated group (n=21 patients); CG=control group (n=5 patients); * p=0.01 (Fisher’s exact test): side effects were more frequently reported in treated patients than in control group, but there was no significant difference in the frequency of which each side effect was reported in both groups.
